# *“It’s really about something! I can save the patient a lot of suffering if I get him to do it”*: between care and coercion in the use of non-invasive ventilation

**DOI:** 10.1186/s12913-025-13621-9

**Published:** 2025-12-23

**Authors:** Anna-Henrikje Seidlein, Susanne Jöbges

**Affiliations:** 1 Institute of Ethics and History of Medicine, University Medicine Greifswald, Greifswald, Germany; 2 Department of Anesthesiology and Intensive Care Medicine (CCM | CVK), Charité Universitätsmedizin, Berlin, Germany

**Keywords:** Non-invasive ventilation, Coercion, Decision-making, Humanizing, Physical restraint

## Abstract

**Background:**

Despite clear evidence that non-invasive ventilation (NIV) is important and effective, both staff and patients report challenges. Patients often perceive NIV as unpleasant, and their acceptance is poor. For healthcare professionals, the patient’s insufficient adherence is demanding and requires considerable expertise, personnel and time resources. Therefore, it is likely that actions are taken to influence the patient’s will in favour of NIV in order to apply it. However, it is not yet clear what these actions look like and what they mean for patients and team members.

**Methods:**

Qualitative semi-structured online interviews with nurses and physicians practicing at German intensive care units have been conducted. Transcribed interviews were analysed using a thematic analysis.

**Results:**

The NIV was described as a frequent and, at the same time, stressful and morally distressing situation in which the patient’s will is deliberately influenced and/or overridden. Three themes were developed inductively from the data that relate to influencing the patient’s will regarding NIV: (1) Diverging professional perspectives on NIV, (2) strategies for carrying out NIV and (3) being caught in a dilemma.

**Conclusion:**

While NIV is an important and evidence-based intervention, its application can lead to moral conflict and both informal and formal coercion if not handled with sensitivity. Promoting respectful treatment, shared decision-making and team training is essential to ensure NIV is experienced as supportive rather than coercive.

**Clinical trial registration number:**

Not applicable.

## Background

Non-invasive mechanical ventilation (NIV) is used as standard in the intensive care unit (ICU) to treat acute respiratory failure, prevent cardiopulmonary complications and improve lung ventilation with a reduction of atelectasis in the postoperative phase [1–4]. The NIV, especially continuous positive airway pressure (CPAP), can improve oxygenation [5], and reduce the frequency of intubations and nosocomial infections. Additionally, it aids ventilator weaning [6]. The application of NIV via nasal, oronasal or helmet interfaces is included in many standards and guidelines for different clinical situations [1, 7, 8] and, therefore, often embedded in the standard treatments and daily routines in the ICU. However, it can be very demanding in terms of personal and time resources and can, thus, also be perceived as a considerable stressor by nurses [9]. The treatment places high demands on the expertise of nursing professionals [10–12]. Furthermore, communication and trust are both a necessary prerequisite and a key challenge to its application [13].

From the patients’ point of view, the use of NIV is unpleasant [14] and associated with negative memories [15]. Experiences such as anxiety, claustrophobia and reactivation of post-traumatic experiences are described [8, 16] as well as facial pressure injury caused by (poorly fitting) interfaces [17–20]. All these factors make it difficult for patients to tolerate NIV. These unpleasant experiences and the situation of a life-threatening illness with limited communication capabilities and cognitive impairments and several other factors can challenge treatment compliance [21].

Successful NIV therapy depends on several psychological factors [22], requires cooperation and adherence, and is an interplay between nurses and patients [23].

Sedative medication can be used to support patients to relieve anxiety and breathlessness during NIV [24], however, it should not be a necessary condition.

There have been sporadic studies on the topic of influencing patients to cooperate with NIV therapy.

**Fig. 1:**
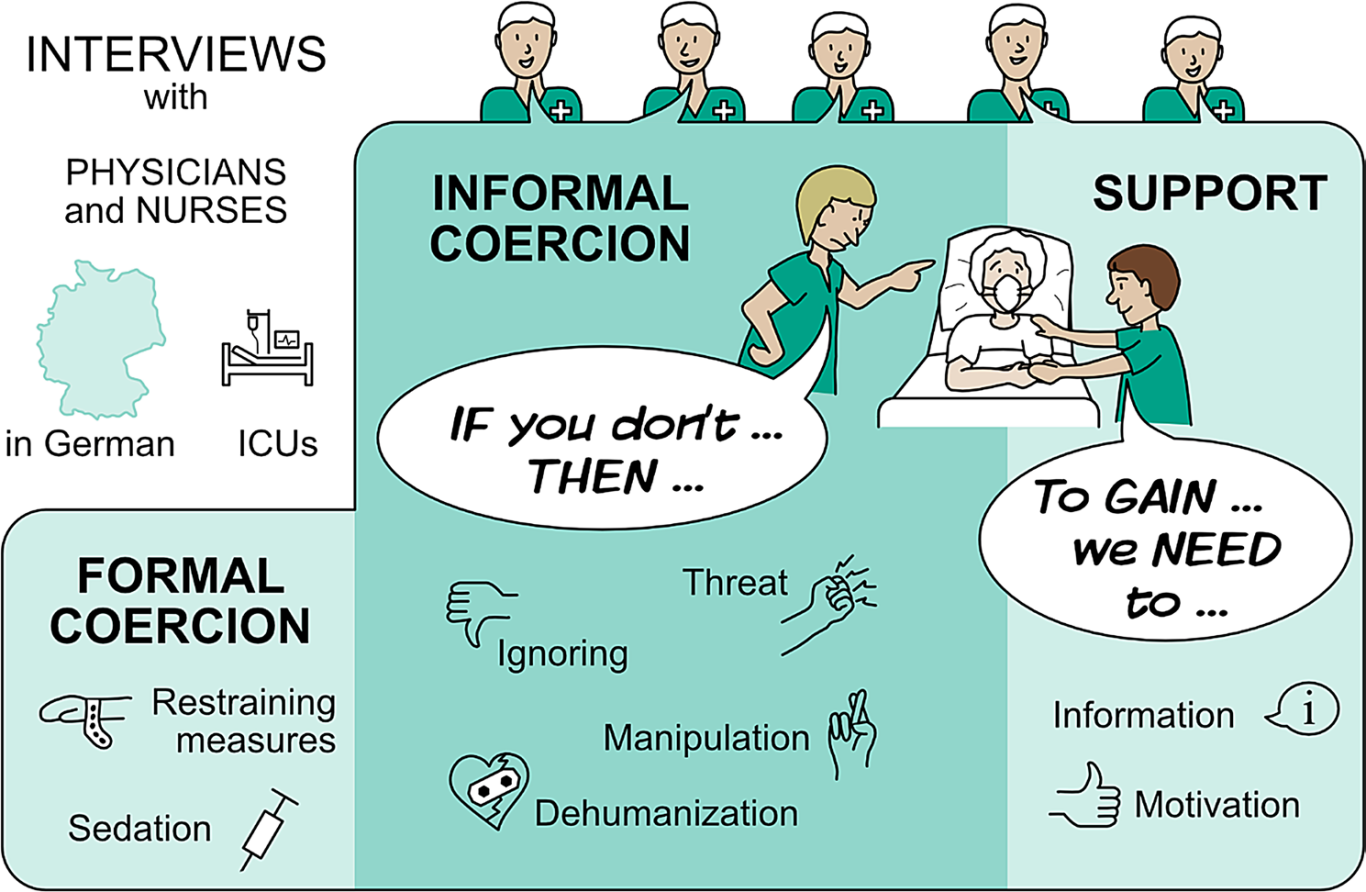
Forms of influencing the patient’s will in the ICU according to [25]

The results presented here on the influence of the team in the context of NIV therapy are part of a larger study whose primary aim was to gain a deeper understanding of informal coercion in the ICU [25], see also Fig. 1].

## Methods

The reporting of our study is in accordance with the COREQ criteria [26].

### Research questions and/or objectives

The results reported here are part of a larger qualitative exploratory study that focused on nurses and physicians’ perceptions of their influence on the patients’ will in the ICU. The results, which describe the possibilities and forms of influencing the patient’s will in general, are published elsewhere [25]. The analysis revealed that many of the situations and problems described by the participants are centred around the topic of NIV. These results were deemed so important that a separate publication is dedicated to them.

### Setting

The study was conducted with nurses and physicians practicing at hospitals in different ownership structures (public, private, non-profit) and of different ICU size (between up to 10 – more than 40 beds) as well as type (surgical, internal, mixed, neurological) in Germany.

### Ethical approval

The approval for this study was obtained from the institutional review board at University Medicine Greifswald, Germany (No.: BB 049/22). Participants were provided with written information outlining the studies objectives, methodology and voluntary nature. Informed consent was obtained from each participant.

### Participants

To recruit interview participants, we distributed study posters to colleagues in the Ethics Section of the German Interdisciplinary Association for Intensive Care and Emergency Medicine, asking them to spread these materials in their intensive care units. A convenience sample of nurses and physicians was recruited via these posters in their wards. Those interested could use the information provided there to obtain further information and arrange an interview appointment. A total of 20 different intensive care units were represented by our participants. All individuals who contacted the study team and subsequently received further information actually participated in the study; no one declined. Physicians and nurses with different qualifications, clinical functions and levels of experience were eligible if practicing for at least 12 months in the ICU. The sample size was determined by theoretical data saturation. (i.e. the point at which additional data only confirms what is already known from the previous interviews and no new information is generated).

### Data collection

Data were collected through semi-structured interviews by SJ (researcher) and AHS (researcher) between September 2022 and February 2023.

The interview guide was developed based on extensive literature review and our previous research. This study used an interview guide developed for a research project entitled ‘Influencing the Critically Ill Patient?‘. It has already been published [25]. Interviews were conducted via videoconferencing, audio recorded and transcribed verbatim.

### Data analysis

Interviews were inductively coded line by line by XX and XX using thematic analysis [27] in MAXQDA software version 2022. All findings were compared and discussed in regular meetings until a shared coding system was reached, which was subsequently worked on further.

## Results

The interviews lasted between 25 and 83 min, with an average length of 41 min. The sample (Table 1) consisted of 14 nurses and 16 physicians. (The names used in this manuscript are anonymous codes for the participants.)

**Table 1:** Sample characteristics; ICU = intensive care unit [25]

	Nurses (*n* = 14)	Physicians (*n* = 16)
**Gender**		
Male	6	12
Female	8	4
**Age** range	24–64 years	29–65 years
(Mean ± SD)	(40 ± 12.7)	(44 ± 9)
**Professional experience** range	1–32 years	3–39 years
(Mean ± SD)	(44 ± 11)	(18 ± 9)
**Professional experience in Intensive Care** range		
(Mean ± SD)	1–26 years	2–25 years
	(12 ± 9)	(10 ± 7)
**Additional qualifications** (n=)		
Specialised intensive care nursing/medicine		
Palliative care	7	10
Emergency medicine	-	2
	-	7
**ICU size** (n =)		
Up to 10 beds	1	1
Up to 20 beds	9	9
Up to 30 beds	2	3
Up to 40 beds	2	1
More than 40 beds	-	2
**Type of ICU** (n =)		
Surgical	6	6
Internal	2	1
Mixed	4	8
Neurological	2	1
**Hospital ownership** (n =)		
Public	9	11
Private (for-profit)	3	4
Non-profit	2	1
**Role**	Head nurse *n* = 2	Resident *n* = 2
	Advanced practice nurse *n* = 1	Consultant *n* = 2
		Senior physician *n* = 9
		Chief physician *n* = 3

Three themes were developed inductively from the data that relate to influencing the patient’s will regarding NIV.

### Diverging professional perspectives on NIV

The interviews revealed that there are different perceptions on the use of NIV, particularly CPAP, within the team, resulting from the unique perspectives, responsibilities and experiences: While physicians determine the indication for NIV, nursing staff are responsible for its implementation. From a nursing perspective, NIV is characterised by the need for constant physical presence and individual care at the bedside. It is very time-consuming, demanding and requires a high level of personnel commitment.

*Well, NIV, that needs strong, close psychosocial support from the carers […], that’s time-consuming; it needs someone to stand next to them for a while and deal with them and adjust things well with them. (Florian, nurse)*

*If doctors worked more closely with patients a few times, I think they would gain a different perspective on what it actually means to be there, that is, what kind of power it exerts and provides. (Emily, nurse)*

Because of the different perspectives within the team, compliance and patient’s will are interpreted very differently, which can also lead to interprofessional conflicts. When interpreting the patient’s verbal and nonverbal signals, nurses often question whether NIV is in line with the patient’s treatment preferences.

*[…] sometimes I also felt like I was left alone. I thought, I’m here with the patient now, the patient is refusing. We’ll have to think again together about how it can work better or something. But no, he has to do CPAP. Full stop. (Emily, nurse)*

*We nursing staff understood this very clearly and the attending senior physician understood this literally and said that the patient did not want to be intubated, but she did want to be ventilated. […], the patient had really resisted the whole time, tried to tear off the mask, we had to restrain and later sedate her. (Vincent, nurse)*

### Strategies for carrying out NIV

The interviewees repeatedly reported difficulties in carrying out NIV. Ways of exerting influence to secure NIV/CPAP range from motivation through convincing to subordination and control, with each form of influence applied through distinct actions.

### Controlling the will

Controlling the will or deliberately overriding it to deliver NIV/CPAP is exercised through physical and/or chemical restraints.

*Because they then resist and, of course, it’s clear that if you’re given a positive pressure mask, […] that I don’t take the will of my patients into account, but rather* ***make*** *the patients* ***compliant*** *with morphine or something like that, so that they are more likely to be immobilised in order to follow the therapy. (Jan, nurse)*

### Subordination

Three strategies, respectively, psychological measures, were identified to reach subordination.

i) *Persuasion*:

*That the patient does not want this procedure, we, yes, I, try to persuade them or convince them that it would be the right thing to do. (Sebastian, physician)*

ii) *Exploiting the professional role*:

*Here too, of course, I’m going to say, yes, you’re absolutely right, it’s a bit like that. I do force them and take advantage of my position as an older, experienced senior doctor, so to speak. They listen to me better than they do to the young doctors or nurses; they’re [*young doctors or nurses*] more likely to give in or allow themselves to be brushed aside. I don’t do that. That’s how it works for us. I’m the last person to talk sternly to the patients and things usually work out.* (*Jacob, physician*)

*It’s usually the case that the nursing staff then come to me and say: Listen, the patient doesn’t want any of this. And then, as a doctor, I try to go back and say: Come on, this is necessary.* (*Anna, physician*)

iii) Threat:

*It’s a bit threatening. You try to describe a scenario to the patient: if you don’t tolerate this mask now, then you’re in for worse. And there are some patients where I choose very clear words because I know that they will get through, and there are many patients where you can’t do that because it scares them off. (Aaron, physician)*

### Convincing manipulative

Three strategies (deception, interpersonal leverage and lies) which can be understood as manipulative convincing of the patient are applied in practice according to the interviewees:

i) Deception.

*Yes, I’ve already experienced it. If you don’t put the mask on now, then you’ll die. Yes, so if-then, that’s a threat. You just have to package it differently, then it’s a conviction. (Ben, physician)*

ii) Interpersonal leverage (i.e. involving close friends and family members to influence patients without their knowledge).

*[…] by getting the family involved, so that they influence the patient to go along with the whole thing. (Lina, physician)*

iii) Lies.

*[…] are forced to undergo prophylactic mask CPAP. Then they’re simply told that they’ll get pneumonia and die if they don’t do it and, hey presto, it’s done. And the patient has to comply because they simply believe it, even if it’s not true […]. (Finn, physician)*

### Convincing argumentative

As described in the interviews, enabling patients to *understand* the need for NIV and then getting them to adhere to therapy can be very challenging. Therefore, a common strategy is trying to find out what the patient has already understood, repeatedly providing them with information, and educating them about the benefits and necessity of NIV in different ways.

*[…] patient who needed NIV and who said quite clearly, no, she didn’t want it. […] and then I sat down on her bed and explained to her what it actually was and told her why she was here in our intensive care unit, and then I also explained to her how I would approach this NIV again and I would be very happy if she gave it another chance, because it would have a significant impact on her health. (Aurelia, nurse)*

### Motivation

In order to motivate patients to use NIV, information is not only provided, but nurses and physicians attempt to tie it in with knowledge about the patient’s life and create a positive outlook concerning people and life events that are relevant to them.

*I always try to motivate patients who are able to communicate and who I, at least, assume understand what they are being told. In other words, I try to explain the importance and find out what the obstacles are. (Paul, physician)*

*[…] then I talk to him, yes, about, I know, his wife or his grandchildren, what’s important to him right now, and when we’ve agreed that I’m not a threat to him after all, yes, and then I say, well, I’d recommend that we put the mask on for ten minutes. Look at your watch. Yes, I’m negotiating with these people. (Vincent, nurse)*

The forms of influence described are not used as different escalation levels, but also in parallel, as the following quote impressively shows:

*Breathing training. […] patients on our ward are usually* ***made to do this by force*** *[…] Sometimes the force is* ***only verbal***, *the nurse shouts at them, intimidates the old granny and says, this is being done now because it’s prescribed and otherwise you’ll get pneumonia. Then the wrists are restrained and the CPAP mask is strapped on. That’s the reality in intensive care units. (Finn, physician)*

### Being caught in a dilemma

There are also different interpretations of the need for NIV between nurses and physicians. When patients refuse NIV, it creates a moral dilemma for nurses. Nurses often find themselves caught between caring, preventing harm and respecting autonomy when they should apply NIV.

*The application of a non-invasive ventilation mask, where you then really force the patient to undergo such a procedure with several people for the patient’s presumed well-being, because you have no chance at all of finding out the patient’s will, because the patient cannot judge this at all. I always find that these are formative situations, where you really, yes, rush in with several people on such a patient […]. (Aaron, physician)*

*[…] by simply starting CPAP training. Of course, I’ve disregarded the patient’s wishes to a certain extent, but I also have the ultimate goal in mind. (Charlotte, nurse)*

Another dilemma arises in the interpretation between the patient’s current will and previously expressed will concerning NIV and ventilation in general. Interviewees described that even if they act against the will of the patient, they often received gratitude afterwards, meaning that they do not know when it is appropriate to override the patient’s will and when it is not.

*[…] afterwards she thanked the senior physician for being so consistent. But I still get goose bumps today, because it was actually against, well, it was actually described quite differently, but because she survived it, she retroactively thanked him for not adhering to her will, or that he had, in fact, recognised her will. But it was a very difficult situation for us carers at the time. […]. (Vincent, nurse)*

### De-escalation strategies

Nurses and physicians describe a variety of strategies to enable NIV. Besides information to educate patients about the disease, reasons for and effects of NIV, benefits are explained to them, and advice is given to motivate them (see also category 2).

The search for compromises and alternatives by nurses plays an important role in this process. It often involves negotiations and concessions on both sides. Alternatives include more and/or longer mobilisation and frequent breathing exercises. These are presented and given to the patient to choose from, giving them the opportunity for participation and self-determination.

*Okay, if you’re supposed to do CPAP training once per shift, you can say, okay, no, to accommodate him, we’ll skip this once […] and do two out of three CPAP training sessions in any case. (Charlotte, nurse)*

*[…] try to accompany that, so I try, if it’s possible, especially when you’re doing CPAP therapy for the first time, sometimes you’re at the bedside for 15–20 min, and that’s perfectly okay. (Henry, nurse)*

*[…], then there is always the option of mobilising him to the edge of the bed if necessary, so that he can breathe better, so, I always give alternatives. (Olivia nurse)*

If these attempts remain unsuccessful, sedation will also be considered in agreement with the patient as a relief option to enable NIV:

*We are now only doing half an hour of NIV, not an hour as usual *[…]* I can give you a drug to at least keep you going for half an hour.* (*Jan, nurse*)

*You get a little morphine to make it more pleasant and easier to bear, and if it doesn’t work, then you don’t have to bear it either, but in small steps, so to speak.* (*Alice, physician*)

*The patient was then given a drug and then he was in a twilight sleep, very relaxed and the CPAP mask could then be put on for at least half an hour or so*. (*Ava, nurse*)

However, it should be noted that there is a very fine line between sedation to relieve the patient and sedation to make the patient compliant through pharmacological measures.

## Discussion

Although the interview guide was not meant to be centred around NIV but primarily on the possibilities and forms of influencing the patient’s will, the use of NIV and measures to achieve adherence were discussed in almost all interviews (28/30). The relevance of this topic for the interviewees led us to dedicate a separate publication to this data. When using NIV at the ICU, various forms of influence are applied, some of which can be categorised as informal coercion. Coercive measures related to ventilation therapy are remembered by patients for a long time [28]. Three points need to be discussed in connection with NIV and influences on the patient’s will.

*Firstly*, NIV seems to be very well integrated into clinical practice in intensive care. The regular use of NIV and the need for and importance of these interventions were described by all respondents based on the evidence available. The interviewees repeatedly mentioned NIV or CPAP interchangeably, described both prophylaxis and therapy, which leads to an unclear context in which NIV is used in some cases. However, the medical requirement recommendations for NIV are often at odds with the experiences of teams with NIV. The experiences also differ greatly between nurses and physicians and can lead to conflicts. Nurses interpret the patient’s current wishes often on the basis of verbal and nonverbal signals and tend to question the appropriateness and usefulness of CPAP therapy. In particular, nurses generally report more negative feelings and regrets about the use of NIV compared to intensivists [23]. It would be beneficial if physicians were more involved in the direct application of non-invasive ventilation (NIV). This would allow them to properly consider the frequent rejection of NIV by patients. The NIV implementation is strongly prioritised both as prophylaxis and therapy but means a high use of resources in practice [10–12]. If compliance with standards and routines is prioritised over the individuality of the patient, this can lead to dehumanisation (that the patient is no longer perceived as a person in the context of routine procedures) or feelings of being a burden for the patient [29].

*Secondly*, influence is used in the ICU to achieve compliance with NIV. The different forms of influence are recognised and reflected by the team. The measures include treatment pressure, informal coercion and restrictive measures (physical, pharmacological coercion). However, motivation and persuasion are also mentioned as options for treatment compliance. The prevalence of physical restraints in the ICU has been described as around 46.7% in previous studies [30] and patients confirm that they have experienced formal coercion [31–33]. By contrast, the use of communicative strategies to influence patients and improve compliance with the recommended treatment has so far only been described in psychiatry. These influencing strategies (informal coercion) include measures aimed at subordination, such as threats, lies and persuasion [34, 35]. Strategies such as deception and “reference to rules and routines” are described in studies by Rugkasa et al. [36] and Pelto-Piri and colleagues [37] from the perspective of healthcare professionals in psychiatry.

All of these measures to influence NIV treatment were mentioned and reflected upon by our interviewees. Many team members expressed moral concerns in context with formal and informal coercion. Such moral conflicts arising between beneficence and autonomy can lead to moral distress and, as a result, to poor patient care and an increased exit from the profession [38, 39].

Thirdly, the team member’s own morality and awareness of the patient as an individual person lead to the search for ways and strategies to deal with the need for NIV therapy. Nurses and physicians describe a variety of strategies to enable NIV. For many team members, motivating and involving the patient is a good way to achieve treatment adherence with NIV. Trust and reliability are seen as key elements for the success of NIV, which is why caregivers describe building a trusting and personal relationship with the patient. They do this by adhering to agreements (e.g. time limits for NIV), emphasising their presence and providing resources to reduce anxiety, and to comfort and distract the patient. In order to make NIV easier for patients, some team members describe the possibility of sedation. Monitored sedation as part of NIV therapy may be beneficial for a selected group of patients with a high risk of NIV intolerance [40]. However, the fine line between facilitating the treatment measure and sedation to control the patient’s behaviour must be noted. Medication outside of a sensible treatment programme constitutes pharmacological restraints [41].

The analysis of the interviews raises the central question of how patients can better participate in decision-making and both formal and informal coercion be prevented regarding NIV.

Therefore, clinical guidelines should not be regarded as absolute mandates [3].

The application of NIV requires careful, individualised clinical judgment. Moreover, the implementation of specific guidelines on managing coercion can support ICU teams in navigating ethically challenging situations in ways that prioritise patient well-being [42–44]. In addition, targeted training on both formal and informal coercion, along with improved team communication, is essential to enhance awareness and the appropriate handling of influencing the patients will and their autonomy [45].

Furthermore, the therapeutic relationship between the patient and the ICU team is a critical factor in the patient’s experience of dependency, particularly in terms of whether influence is perceived as coercive [46, 47]. In the context of intensive care medicine, where patients are often in highly vulnerable states, it is imperative for staff to reflect on their own positions of power. Research shows that the behaviour of ICU clinicians can contribute to patients and families experiencing a sense of dehumanisation during care [48].

In order to support patients effectively during NIV, it is essential to establish a trusting therapeutic relationship that facilitates shared decision-making and patient participation in the treatment process [49, 50]. Many ICU professionals actively seek strategies to engage and motivate patients during NIV therapy; in this regard, involving family members can be a valuable resource [8, 51]. Innovative approaches, such as the use of music or virtual reality during NIV, have also shown promise in enhancing patient comfort and acceptance [52].

Although there are recommendations and guidelines for NIV, these do not always align with the team’s practical experience in its use. Instead of mechanically following these guidelines, treatment should be selected for and adopted to the individual patient. For instance, high-flow nasal therapy can be used as an alternative to stabilise oxygen levels. Offering patients therapy to stabilise their oxygen supply without coercion remains a key ethical priority.

Intensive care requires a “humanized ICU” grounded in an ethos that respects patient autonomy. However, the interviewees’ responses indicate that this principle is not always upheld in clinical practice, which is ethically and legally unacceptable and must be addressed.

In summary, ICU teams possess both the responsibility and the opportunity to critically reflect on and intentionally choose the forms of influence they employ when administering NIV. Humanising the ICU involves more than technical excellence. It requires a commitment to respecting the patient’s dignity, autonomy and individual needs throughout the entire course of care and treatment, also including NIV.

Limitation: Not only important treatment decisions, but also NIV, were found to be associated with difficult situations and negotiation processes. This publication is limited to presenting descriptive data. When interpreting the results, it should be considered that convenience sampling was used as a recruitment strategy.

Another limitation is that the study was conducted in German intensive care units. Further studies in an international context are needed to evaluate statements regarding the use of informal coercive measures.

## Conclusion

Although there is robust evidence and a range of established standards supporting the use of NIV, both ICU teams and patients report significant challenges in its application. These challenges include the perception and experience of treatment-related pressure. Such dynamics can place considerable emotional and moral burden on staff, contributing to moral distress.

**Fig. 2:**
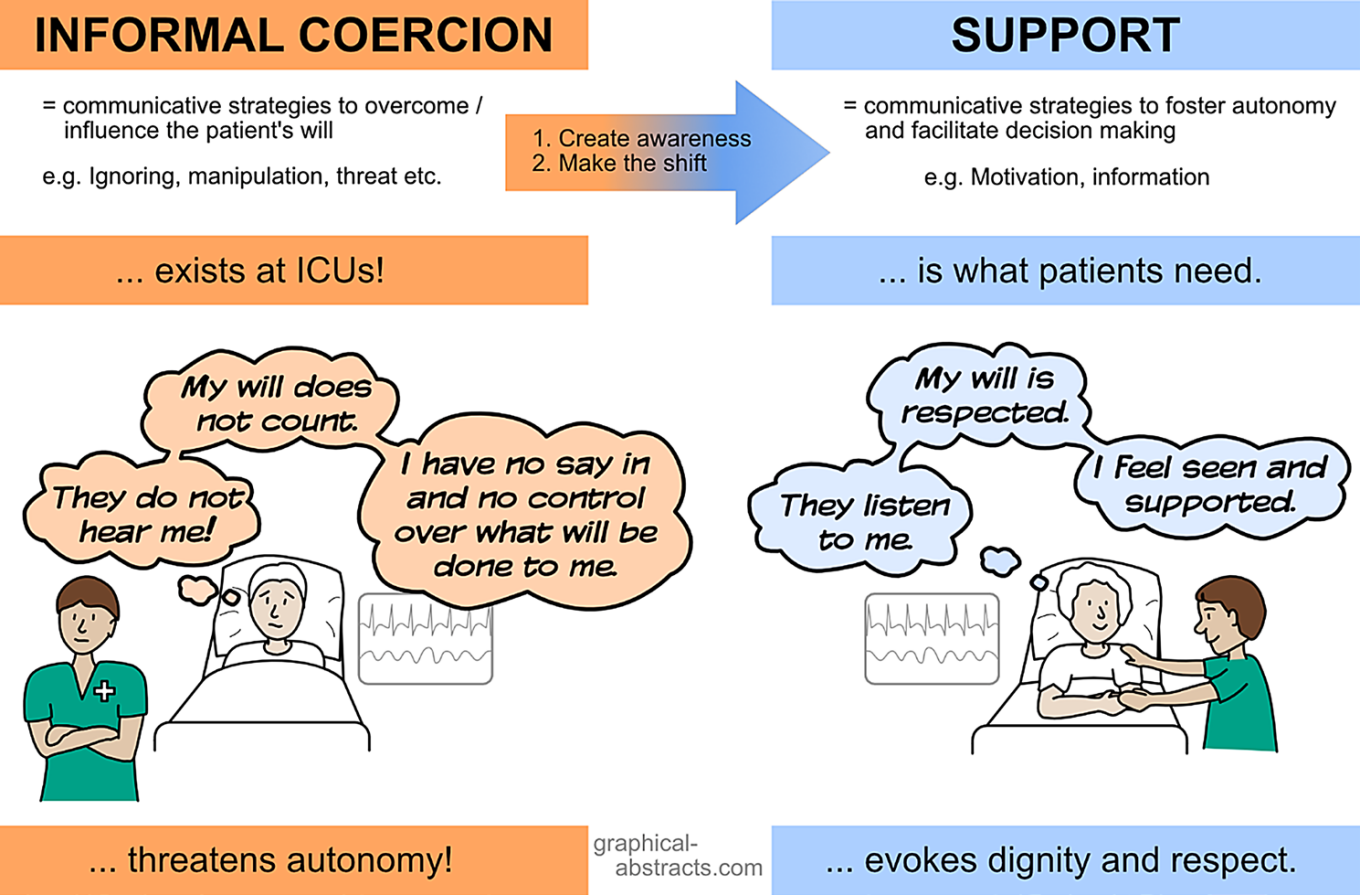
What is needed: The shift from informal coercion to support

From the patient’s perspective, NIV is sometimes recalled as a coercive experience, particularly within the context of dependency in the ICU. The key factor in distinguishing between supportive care and coercion lies in how the patient perceives their dependency, the degree of respect conveyed by the ICU team and the extent to which shared decision-making is facilitated. Addressing these issues effectively to create a shift from informal coercion to support (Fig. 2) when applying NIV requires targeted training, a reflective team culture and a nuanced understanding of each patient’s individual situation.

## Data Availability

The anonymized dataset analysed during the current study are available from the corresponding author on reasonable request.
